# Improving adherence to venous thromoembolism prophylaxis using multiple interventions

**DOI:** 10.4103/1817-1737.78425

**Published:** 2011

**Authors:** Jaffar A. Al-Tawfiq, Bassam M. Saadeh

**Affiliations:** *Internal Medicine, Dhahran Health Center, Saudi Aramco Medical Services Organization, Saudi Aramco, Dhahran, Saudi Arabia*

**Keywords:** Deep vein thrombosis, quality improvement, thromboprophylaxis, underutilization, venous thromboembolism

## Abstract

**OBJECTIVE:**

In hospital, deep vein thrombosis (DVT) increases the morbidity and mortality in patients with acute medical illness. DVT prophylaxis is well known to be effective in preventing venous thromoembolism (VTE). However, its use remains suboptimal. The objective of this study was to evaluate the impact of quality improvement project on adherence with VTE prophylaxis guidelines and on the incidence of hospital-acquired VTEs in medical patients.

**METHODS:**

The study was conducted at Saudi Aramco Medical Services Organization from June 2008 to August 2009. Quality improvement strategies included education of physicians, the development of a protocol, and weekly monitoring of compliance with the recommendations for VTE prophylaxis as included in the multidisciplinary rounds. A feedback was provided whenever a deviation from the protocol occurs.

**RESULTS:**

During the study period, a total of 560 general internal medicine patients met the criteria for VTE prophylaxis. Of those, 513 (91%) patients actually received the recommended VTE prophylaxis. The weekly compliance rate in the initial stage of the intervention was 63% (14 of 22) and increased to an overall rate of 100% (39 of 39) (*P* = 0.002). Hospital-acquired DVT rate was 0.8 per 1000 discharges in the preintervention period and 0.5 per 1000 discharges in the postintervention period, *P* = 0.51. However, there was a significant increase in the time-free period of the VTE and we had 11 months with no single DVT.

**CONCLUSION:**

In this study, the use of multiple interventions increased VTE prophylaxis compliance rate.

Development of deep vein thrombosis (DVT) in hospitalized patients is associated with higher risk for adverse outcomes, longer hospital stay, and increased mortality. It is estimated that 5–10% of all deaths in hospitalized patients are accounted for by pulmonary embolism and thus venous thrombo-embolism (VTE) is considered the most common preventable cause of in-hospital death.[1,2]

Acute and chronic illnesses increase patient’s predisposition to VTE. However, many studies showed that a high percentage of hospitalized patients do not receive adequate VTE prophylaxis. In addition, evidence-based consensus guidelines are available for such patients. In one study, 51.8% of all at-risk patients received ACCP-recommended VTE prophylaxis (54.7% of surgical patients, 32.5% of medical patients).[3] A community-wide study of 16 hospitals in USA showed that VTE prophylaxis was provided for only 32% of patients at high risk.[4] Studies were conducted to evaluate the available options to improve the compliance rate with VTE prophylaxis.[5,6] The primary objective of the study was to examine the impact of multi-interventions on the rate of compliance with VTE prophylaxis in medical patients from June 2008 to May 2009. The secondary objective was to compare the rate of VTE before and after the implementation of these multi-interventions.

## Methods

All admitted medical patients were assessed for VTE risk in accordance with the 2004 ACCP guidelines.[1] The risk for VTE was considered in the presence of predisposing conditions or clinical characteristics. Predisposing conditions include acute infectious disease, congestive heart failure (New York Heart Association class III or IV disease), acute respiratory disease, and malignancy.[1] The clinical characteristics included previous venous thromboembolism, older age (especially >75 years), immobility or paresis, obesity (BMI >30 kg/m^2^), hormone therapy, or pregnancy.[1] We defined VTE prophylaxis compliance as any type of prophylaxis (mechanical or pharmacologic) as indicated according to the ACCP guidelines.[1] The recommendations of the ACCP were also followed in regards to the drug choice, dose, duration, and the timing of the prophylaxis. Contraindications for pharmacologic anticoagulation were as follows: if there is a risk of excessive bleeding, such as with recent gastrointestinal bleeding, hemorrhagic stroke, or hemostatic defects such as severe thrombocytopenia (platelets <50,000 mm^3^), history of heparin-induced thrombocytopenia (HIT), active major bleeding, uncontrolled hypertension (systolic >200, diastolic > 120).[1]

### Design

Information about the adherence to VTE prophylaxis was collected on weekly basis. The physicians were informed by e-mail when VTE prophylaxis was not prescribed. This is served as an initial intervention, followed by a second intervention of assessing the indication and prescription of VTE prophylaxis for the medical patients in the weekly multidisciplinary rounds. In addition, a formal education of physicians was given on VTE prophylaxis guidelines and later a medical services policy was developed.

The data were presented as the compliance to the recommended prophylaxis by dividing the number of those who received the recommended prophylaxis by the total number of eligible patients for VTE prophylaxis on each week. We also calculated the rate of in-hospital acquired VTE and the rate of occurrence of VTE per 1000 discharges. The compliance rate before and after the implementation of the multi-intervention strategy was compared using the χ^2^-test and the *P* value of ≤0.05 was considered statistically significant.

## Results

During the study period from June to December 2008, a total of 560 general internal medicine patients met the criteria for VTE prophylaxis and those were included in the study. Of those, 513 (91%) patients actually received the recommended VTE prophylaxis. The majority of the patients (95%) received subcutaneous heparin and the remaining patients received nonpharmacological therapy.

**Figure 1:**
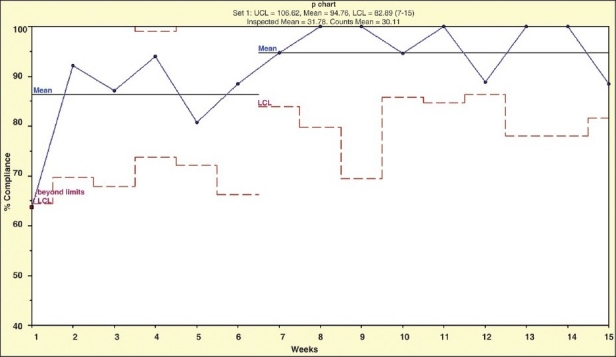
A run chart showing the weekly compliance rate with DVT prophylaxis. The X-axis shows the time of the study (weeks) and the Y-axis shows the compliance rate. The dotted lines show the lower control limits and the straight lines show the mean compliance rates at the beginning and end of the study

The base line data for VTE prophylaxis indicated about 65% compliance in the preceding three months. In the initial 2 weeks of the intervention, the weekly compliance rate was 63% (14 of 22). However, the compliance rate increased to an overall rate of 100% (39 of 39) (*P* = 0.002) [Figure 1] by week 14 of the study. Hospital-acquired VTE rate was 0.8 per 1000 discharges in the preintervention period of the preceding 10 months (95% CI: 0.10–1.53) and 0.5 per 1000 discharges in the postintervention period (95% CI: 0.01–1.05), *P* = 0.51 of the study duration from June to December 2008. However, there was a significant increase in the time-free period of the VTE and we had 11 months with no single VTE.

## Discussion

In this study, we took a quality improvement initiative to increase the compliance rate with VTE prophylaxis in medical patients. At the beginning of the study, underutilization of VTE prophylaxis (63%) was observed. This low rate is similar to previously reported rates from different parts of the world.[5,7,8] In those studies, the overall compliance with VTE prophylaxis ranges from 26% to 51%.[5,7,8] In a recent study from the Epidemiologic International Day for the Evaluation of Patients at Risk for Venous Thromboembolism in the Acute Hospital Care Setting (ENDORSE) trial, 54.7% of surgical patients and 32.5% of medical patients received ACCP-recommended VTE prophylaxis.[3] In another ENDORSE study, 39.5% of at-risk medical patients received the ACCP-recommended VTE prophylaxis.[9]

Underutilization of VTE prophylaxis can be explained by underestimation of the magnitude of the problem and fear of bleeding complications. Several interventions were tested to increase the compliance with VTE prophylaxis. In a study in Ontario, Canada, diagnosis-specific order sets were used to increase compliance with VTE prophylaxis. Patients admitted with order sets were more likely to be ordered VTE prophylaxis than patients admitted with free-text orders (44.0% *versus* 20.6%).[5] In another study of the effect of a CD-ROM-based educational intervention on adherence to VTE prophylaxis guidelines, the rate of compliance increased from 75% before the CD-ROM intervention to 95% after the intervention.[10] In addition, it was shown that implementation of clinical guidelines for VTE prophylaxis through computer-based clinical decision support systems in an orthopedic surgery department and integrated into the hospital information system changed physician behavior and improved compliance with guidelines.[11] In a study from USA, electronic alerts for hospitalized high-VTE risk showed that the majority of alerted physicians in the cohort study did not order VTE prophylaxis despite the alerts. Thus, the finding suggests that other strategies are needed to improve the use of VTE prophylaxis in hospitalized high-risk patients, especially in medical service patients.[12] In another study, a formal CME program significantly increased the frequency with which physicians prescribed prophylaxis for VTE from 29% in 1986 to 52% in 1989 (*P* <0.001).[4] However, we used multiple steps of interventions to provide optimal compliance. We initially used education followed by daily e-mail reminder and eventually, we incorporated VTE prophylaxis in the weekly round. We are now in the process of developing standing orders for VTE prophylaxis.

Similar to another study, we found no significant difference in the rates of DVT and anticoagulant-related adverse events in the two time-intervals.[13] One reason is that the primary goal of this study was to evaluate the effectiveness of the interventions on increasing the rate of compliance with VTE prophyalxis. This process measure is thought to be more appropriate than outcome measures.[13] However, another study showed a lower incidence of clinically diagnosed, objectively confirmed DVT or pulmonary embolism at 90 days between the intervention group and the control group (4.9% vs. 8.2%, *P* < 0.001).[14]

In conclusion, the use of multiple interventions seems to be more effective in improving the compliance with VTE prophylaxis. We used initially passive dissemination of guidelines and this resulted in some improvement in the compliance rate. Subsequently, we used reminders, audit and feedback to facilitate the compliance with the recommendations. With these interventions, the compliance rate increased to 92.8%. Thus, the use of multiple strategies was more effective than a single strategy used in isolation.[6] Other strategies included educational sessions and risk stratification guidelines to improve identification and prophylaxis of medical patients.[15] In a recent paper, it was suggested that embedding a VTE prevention protocol into admission, transfer, and perioperative order sets is a key strategy in the prevention of VTE.[16]
